# Comparison of Clinical Features and Outcomes of Appendectomy in Elderly vs. Non-Elderly: A Systematic Review and Meta-Analysis

**DOI:** 10.3389/fsurg.2022.818347

**Published:** 2022-02-21

**Authors:** Jie Yuan, Qingfeng Chen, Weicong Hong, Lifeng Yu, Xuen Li

**Affiliations:** ^1^Department of General Surgery, Longshan Hospital of Cixi City, Ningbo, China; ^2^Department of Gastrointestinal Surgery, Ningbo First Hospital, Ningbo Hospital of the Zhejiang University, Ningbo, China

**Keywords:** appendectomy, laparoscopic, acute appendicitis, elderly, non-elderly, young, meta-analysis, systematic review

## Abstract

**Background:**

The objective of this study is to compare clinical and surgical outcomes of appendectomy among elderly and non-elderly subjects.

**Methods:**

A systematic search was conducted on PubMed, Scopus, and Google academic databases. Studies, observational in design, that compared peri-and postoperative outcomes of appendectomy, in patients with acute appendicitis, between elderly and non-elderly/younger subjects were considered for inclusion. Statistical analysis was performed using STATA software.

**Results:**

A total of 15 studies were included. Compared to non-elderly patients, those that were elderly had an increased risk of complicated appendicitis [relative risk (RR), 2.38; 95% CI: 2.13, 2.66], peritonitis [RR, 1.88; 95% CI: 1.36, 2.59], and conversion from laparoscopic to open appendectomy [RR, 3.02; 95% CI: 2.31, 3.95]. The risk of overall postoperative complications [RR, 2.59; 95% CI: 2.19, 3.06], intra-abdominal abscess [RR, 1.84; 95% CI: 1.15, 2.96], wound infection [RR, 3.80; 95% CI: 2.57, 5.61], and use of postoperative drainage [RR, 1.14; 95% CI: 1.09, 1.19] was higher among the elderly. The risk of readmission (30 days) [RR, 1.61; 95% CI: 1.16, 2.24] and mortality (30 days) [RR, 12.48; 95% CI: 3.65, 42.7] was also higher among elderly.

**Conclusions:**

Findings suggest an increased risk of peri-and postoperative complications among elderly subjects undergoing appendectomy, compared to non-elderly subjects.

**Systematic Review Registration:**

https://www.crd.york.ac.uk/prospero/, identifier: CRD42021286157.

## Introduction

Acute appendicitis (AA) is among the common clinical conditions encountered in a surgical emergency unit. The global incidence of acute appendicitis is around 10% with the peak during 20–40 years of life (1, 2). Available statistics suggest that around 70% of all cases of AA are under the age of 30 years (2). However, the condition could occur anytime during the life span. With the increase in life expectancy noted in recent years, there has been a shift in the age incidence of AA with an increasing number of elderly subjects being affected. Currently, 5-10% of all cases occur in the elderly population (1, 3). The occurrence of AA among the elderly poses some practical challenges. The typical symptoms are often present in only one-fourth of these subjects and around 30–35% of the diagnosis is made after a significant delay from the time of the onset of symptoms (4–6). Delayed diagnosis increases the possibilities of complications, such as perforation, gangrene, formation of abscess, and peritonitis. Furthermore, elderly subjects are usually frail and weak to undergo emergency surgery and are often met with sub-optimal outcomes post-surgery (4–6).

Despite the non-operative or conservative management being available for AA, appendectomy remains the gold standard for managing acute appendicitis (7). A recent systematic review has shown that laparoscopic appendectomy (LA) is acceptable for the elderly population with favorable outcomes (8). This meta-analysis has noted decreased rates of mortality, postoperative morbidity, and shorter hospitalization among elderly subjects undergoing laparoscopic appendectomy, compared to open appendectomy (8). Even when LA is documented to be acceptable for elderly subjects, for a treating surgeon, it is important to understand whether there are differential outcomes of appendectomy based on the age of the patient. Such information will help the treating surgeon to be more careful, particularly when operating over an elderly subject and in the identification of patients that are more prone to develop peri- and postoperative complications. There have been several studies that have looked at the comparative outcomes among elderly and non-elderly/younger subjects. However, a systematic synthesis of findings from these studies is needed to provide conclusive evidence and, thereby, guide the clinical practice. With this consideration, the current meta-analysis was conducted to compare important clinical and surgical outcomes of appendectomy among elderly and non-elderly subjects.

## Materials and Methods

### Search Strategy

The study processes complied with the PRISMA (Preferred Reporting Items for Systematic Reviews and Meta-analyses) guidelines (9) and were registered at PROSPERO (CRD42021286157). Through the use of electronic search engines—PubMed, Scopus, and Google academic databases—a thorough systematic search of English language papers published until October 31 2021 was carried out. The search strategy included the use of medical subject heading (MeSH) terminology as well as free text words. Details of the search strategy used have been presented in Supplementary Table 1. The literature search aimed at identifying studies that examined the clinical and survival outcomes of appendectomy for acute appendicitis among elderly and non-elderly subjects.

### Selection Criteria and Methods

Upon identification of studies on literature search and removal of the duplicates, two subject experts from the team reviewed the studies and screened the titles and abstracts as the initial step. The full text of possible studies was subsequently reviewed. Any disagreements in the inclusion of the studies were resolved through discussions between the study authors. Only those studies were included in the meta-analysis that fulfilled the inclusion criteria. To identify additional literature, the reference list of the included studies was also reviewed.

#### Inclusion Criteria

Studies that compared clinical and/or surgical outcomes for appendectomy, done with the indication of acute appendicitis, between elderly and non-elderly/younger subjects, were considered for inclusion. Studies that were observational in design—either cohort or case-control or analyzed retrospective data—were considered for inclusion.

#### Exclusion Criteria

Case reports or review articles were excluded. Those studies that did not provide data on the outcomes of interest or those that did not compare outcomes between elderly and non-elderly/younger subjects were excluded.

### Data Extraction and Quality Assessment

Through the use of a pretested data extraction sheet, two study authors separately extracted data from the included studies. Data extracted mainly included the study identifier, i.e., the name of the first author with the year of publication, study setting and design, subject characteristics, sample size, and the key findings. The Newcastle-Ottawa Quality Assessment Scale was used to assess the quality of included studies (10).

### Statistical Analysis

This meta-analysis was conducted using STATA version 16.0. The effect sizes, along with 95% CI, were reported as pooled relative risk (RR) for categorical outcomes and weighted mean difference (WMD) for continuous outcomes. Subgroup analysis was done based on the type of appendectomy used in the study: open, laparoscopic, or mixed (i.e., some subjects received open and some received laparoscopic appendectomy). For the analysis, *I*^2^ was used to denote heterogeneity. In instances where the value of I^2^ exceeded 50%, the random-effects model was used (11). For reporting statistical significance, a *p* < 0.05 was considered. Egger's test was employed to assess for the presence or absence of publication bias (12).

## Results

**Figure 1:**
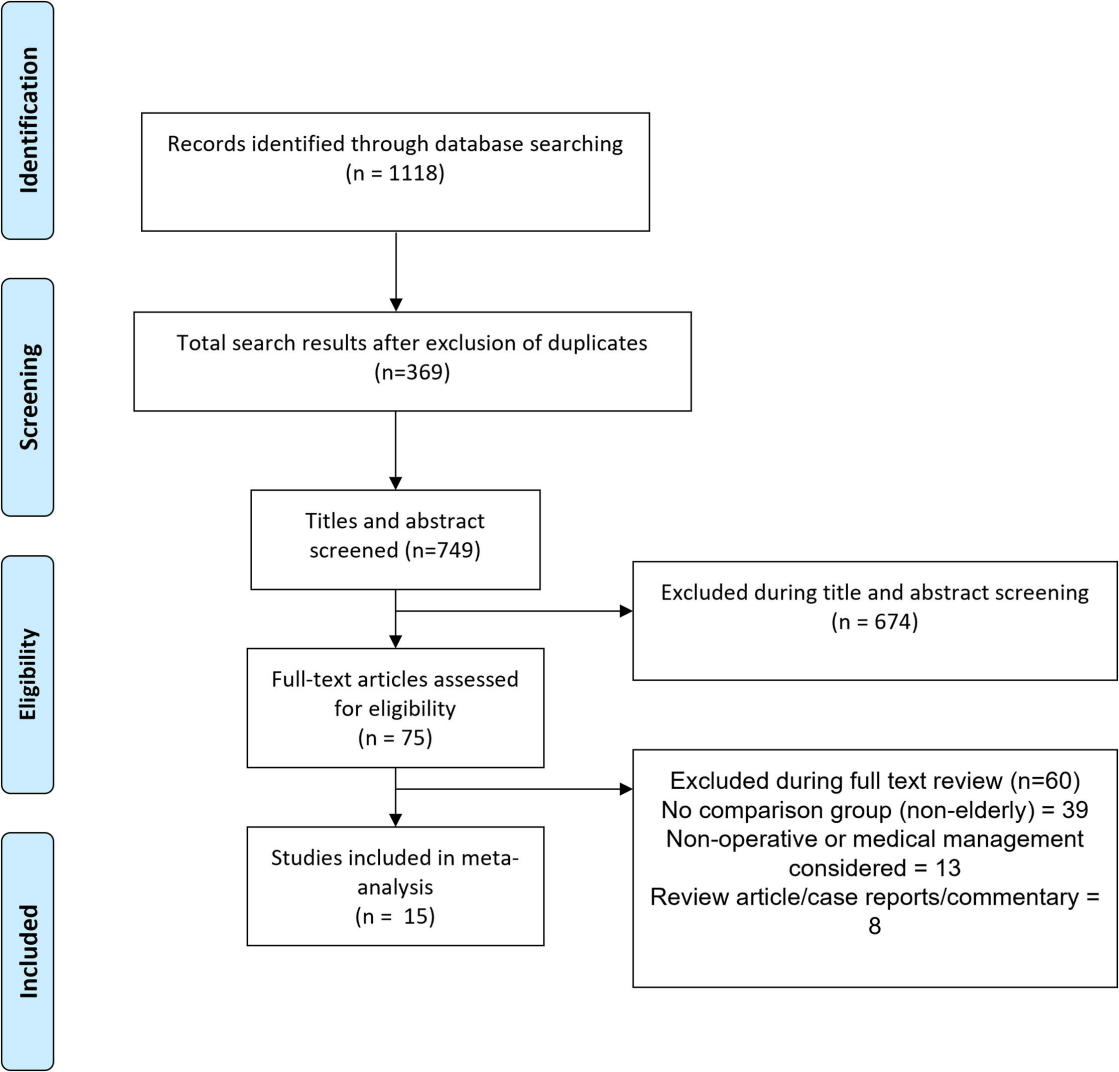
The selection process of the studies included in the review.

Using the search strategy and after removal of the duplicates, overall, 749 citations were obtained (Figure 1). Screening of the titles and abstracts led to the removal of 674 citations. Out of the remaining 75 studies, 60 were excluded after reading the full text. Finally, a total of 15 studies were considered for inclusion (5, 13–26). Table 1 presents the details of the studies included in the review. In one study by Guller et al. (24), the authors presented separate data comparing outcomes in elderly and non-elderly subjects by type of appendectomy (open and laparoscopic). Therefore, for the analysis, this study has been considered as two different studies, and this has been indicated in Table 1 as well. Thirteen of the included studies were retrospective in design. One study was prospective in design and another study used both retrospective data and prospectively collected data. A total of 4 studies were done in the United States, 3 in Israel, and one study was multicentric (conducted across different institutions in Poland and Germany). One study each was done in Argentina, Japan, France, Nepal, Egypt, Poland, and Malta.

**Table 1:** Characteristics of the studies included in the meta-analysis.

**References**	**Study design**	**Country**	**Participant characteristics**	**Age categorization**	**Sample size**	**Key outcomes (comparison group is “non-elderly”)**
Angeramo et al. (13)	Prospective follow up	Argentina	Patients undergoing laparoscopic appendectomy; around 51% male; BMI ≥ 30 kg/m^2^ (5.6%); ASA score of I or II (98%)	Elderly: ≥65 years Non-elderly: <65 years	2009 (elderly: 122; non-elderly: 1887)	*Complicated appendicitis (perforation or gangrene/necrotic)*: RR 2.73 (95% CI: 2.26, 3.32) *Peritonitis*: RR 1.66 (95% CI: 1.43, 1.92) *Readmission*: RR 2.16 (95% CI: 0.94, 4.97) *Postoperative intra-abdominal abscess*: RR 3.76 (95% CI: 1.86, 7.62) *Overall post-operative complication:* RR 2.24 (95% CI: 1.68, 3.01) *Conversion rate* (from laparoscopy to open surgery): RR 2.32 (95% CI: 1.00, 5.36) *Operative time (mean, SD; in minutes):* 65 (34.2) vs. 56 (30.8) *Length of hospital stay (mean, SD; in days):* 3.40 (4.83) vs. 1.62 (4.17) No deaths in either of the two groups
Cohen-Arazi et al. (14)	Retrospective review	Israel	Patients who underwent appendectomy due to acute appendicitis (heterogenous population with some undergoing laparoscopic appendectomy and some open appendectomy); mean age in the elderly group was 74.6 years whereas those in the non-elderly group were in the age range of 20–45 years	Elderly: ≥65 years Non-elderly: <65 years	198 (elderly: 74; non-elderly: 124)	*Complicated appendicitis (perforation or gangrene/necrotic):* RR 3.74 (95% CI: 2.08, 6.73) *Peritonitis*: RR 1.05 (95% CI: 0.36, 3.08) Overall post-operative complication*: RR 6.70 (95% CI: 2.33, 19.3) *Conversion rate* (from laparoscopy to open surgery): RR 3.35 (95% CI: 0.31, 36.3) *Operative time (mean, SD; in minutes):* 88.2 (30.0) vs. 84 (42) *Length of hospital stay (mean, SD; in days):* 6.3 (3.2) vs. 3.4 (1.8) *Use of laparoscopic appendectomy*: RR 0.56 (95% CI: 0.42, 0.74) *ICU admission:* RR 8.37 (95% CI: 1.00, 70.3) *Use of drains*: RR 0.72 (95% CI: 0.19, 2.69) No deaths in either of the two groups *Complications included ileus, post-operative bleeding, pulmonary oedema, surgical site infection, sepsis, acute tubular necrosis, atrial fibrillation and delirium.
Dhillon et al. (15)	Retrospective review	USA	Patients undergoing an appendectomy (specific details on whether LA or open appendectomy not provided): those who underwent an interval appendectomy or an appendectomy for a reason other than appendicitis were excluded; mean age in the elderly group was 67.4 years and in non-elderly group was 37.3 years; males (53.5%); mean BMI of 25.8 kg/m2	Elderly: ≥65 years Non-elderly: <65 years	1,242 (elderly: 52; non-elderly: 1,190)	*Complicated appendicitis (perforation or gangrene/necrotic)*: RR 3.11 (95% CI: 2.28, 4.25) *Length of hospital stay (mean, SD; in days):* 3.8 (3.6) vs. 2.3 (2.0) *Readmission*: RR 1.18 (95% CI: 0.38, 3.65) Mortality*: RR 7.49 (95% CI: 0.31, 181.7) *Only one death in the non-elderly group
Fan et al. (16)	Retrospective review	USA	Patients ≥18 years of age undergoing appendectomy with preoperative imaging consistent with acute appendicitis; ASA score of I or II (83%); male (52%); median age in the elderly group was 71 years and in non-elderly group was 34 years; around 97% had laparoscopic appendectomy and only around 3% had open appendectomy	Elderly: ≥65 years Non-elderly: <65 years	21,586 (elderly: 2060; non-elderly: 19,526)	*Complicated appendicitis (perforation or gangrene/necrotic)*: RR 2.23 (95% CI: 2.01, 2.48) *Peritonitis*: RR 2.33 (95% CI: 2.08, 2.62) *Readmission*: RR 2.14 (95% CI: 1.79, 2.54) *Postoperative intra-abdominal abscess*: RR 1.43 (95% CI: 1.12, 1.83) *Conversion rate* (from laparoscopy to open surgery): RR 2.70 (95% CI: 2.13, 3.42) *Operative time (mean, SD; in minutes):* 50 (6.0) vs. 46 (5.0) *Length of hospital stay (mean, SD; in days):* 2.0 (0.5) vs. 1.0 (0.17) *Use of laparoscopic appendectomy*: RR 0.94 (95% CI: 0.93, 0.96) *Mortality*: RR 2.56 (95% CI: 0.79, 8.25)
Kirshtein et al. (17)	Retrospective review	Israel	Patients undergoing laparoscopic appendectomy due to acute appendicitis; females (~60%); mean age in elderly group was 70.1 years and mean age in young group was 32.7 years; increased use of anti-coagulants in elderly (32.7%) compared to young (1.4%); increased comorbidities in elderly (57.4%) compared to younger group (9.5%)	Elderly: ≥60 years Non-elderly: <60 years	477 (elderly: 54; non-elderly: 423)	*Complicated appendicitis (perforation or gangrene/necrotic)*: RR 2.01 (95% CI: 1.31, 3.11) *Readmission (30-day)*: RR 0.81 (95% CI: 0.26, 2.57) *Postoperative intra-abdominal abscess*: RR 1.96 (95% CI: 0.57, 6.72) *Conversion rate* (from laparoscopy to open surgery): RR 5.59 (95% CI: 1.84, 17.0) *Operative time (mean, SD; in minutes):* 42.5 (10.1) vs. 36.8 (8.7) *Length of hospital stay (mean, SD; in days):* 4.8 (1.7) vs. 2.5 (0.91) Overall post-operative complication*: RR 1.74 (95% CI: 0.75, 4.02) No deaths in either of the groups *includes upper GI bleeding, post-operative fever and abdominal pain, wound infection and other infectious complications.
Lasek et al. (18)	Observation study (both retrospective data and prospectively collected data used)	Multicentric (Poland and Germany)	Patients undergoing laparoscopic appendectomy due to acute appendicitis; males (~52%); median BMI of 26 kg/m^2^; obesity (18%); majority with ASA I and II (95%)	Elderly: ≥65 years Non-elderly: <65 years	4,618 (elderly: 334; non-elderly: 4,284)	*Complicated appendicitis (perforation or gangrene/necrotic)*: RR 1.84 (95% CI: 1.62, 2.08) *Readmission*: RR 1.19 (95% CI: 0.63, 2.25) *Overall post-operative complication:* RR 1.85 (95% CI: 1.34, 2.53) *Conversion rate* (from laparoscopy to open surgery): RR 2.34 (95% CI: 1.74, 3.15) *Need for reintervention*: RR 2.32 (95% CI: 1.35, 3.97) *Operative time (mean, SD; in minutes):* 60 (5.83) vs. 50 (5.0) *Length of hospital stay (mean, SD; in days):* 5 (0.5) vs. 3 (0.3) *Use of drains (post-operative drainage)*: RR 1.14 (95% CI: 1.09, 1.19)
Mima et al. (19)	Retrospective review	Japan	Patients undergoing interval laparoscopic appendectomy due to acute appendicitis; females (~54%); BMI ≥25 kg/m^2^ (28%); comorbidities (8%)	Elderly: ≥70 years Non-elderly: <70 years	47 (elderly: 18; non-elderly: 29)	*Operative time (mean, SD; in minutes):* 57 (8.16) vs. 77 (8.33) *Length of hospital stay (mean, SD; in days):* 15 (1.16) vs. 15 (1.16) *Use of drains (post-operative drainage)*: RR 0.40 (95% CI: 0.05, 3.33) *Postoperative intra-abdominal abscess*: RR 0.53 (95% CI: 0.03, 12.3) No mortality in either of the two groups
Renteria et al. (20)	Retrospective review	USA	Patients undergoing appendectomy due to acute appendicitis; laparoscopic appendectomy in around 91% patients; male (90.7%); mean BMI of 30.3 kg/m^2^; ASA I/II (69.3%); comorbidities (44%)	Elderly: ≥60 years Non-elderly: <60 years	257 (elderly: 62; non-elderly: 195)	*Complicated appendicitis (perforation or gangrene/necrotic)*: RR 1.87 (95% CI: 1.41, 2.47) *Readmission*: RR 1.22 (95% CI: 0.54, 2.79) *Overall post-operative complication:* RR 1.35 (95% CI: 0.54, 3.36) *Conversion rate* (from laparoscopy to open surgery): RR 3.15 (95% CI: 1.05, 9.40) *Operative time (mean, SD; in minutes):* 67 (30) vs. 58.4 (26.2) *Length of hospital stay (mean, SD; in days):* 4.2 (6.5) vs. 1.6 (1.8) *Estimated blood loss (mean, SD; in ml)*: 24 (32) vs. 17.4 (28.1)
Segev et al. (21)	Retrospective review	Israel	Patients undergoing appendectomy due to acute appendicitis; laparoscopic appendectomy in 55% patients; male (55.4%); mean age of 78 yrs in elderly group and 23 yrs in non-elderly group	Elderly: ≥68 years Non-elderly: <68 years	1,898 (elderly: 68; non-elderly: 1,830)	*Complicated appendicitis (perforation or gangrene/necrotic)*: RR 2.52 (95% CI: 1.85, 3.43) *Postoperative intra-abdominal abscess*: RR 0.47 (95% CI: 0.07, 3.36) *Overall post-operative complication:* RR 2.58 (95% CI: 1.81, 3.67) *Post-operative wound infection*: RR 2.41 (95% CI: 1.08, 5.35) *Use of laparoscopic appendectomy*: RR 1.05 (95% CI: 0.85, 1.29) *Conversion rate* (from laparoscopy to open surgery): RR 4.89 (95% CI: 1.73, 13.8) *Operative time (mean, SD; in minutes):* 88 (34.7) vs. 66.9 (24.9) *Length of hospital stay (mean, SD; in days):* 5.7 (4.6) vs. 3.2 (2.8)
Weinandt et al. (22)	Retrospective review	France	Patients undergoing appendectomy due to acute appendicitis; laparoscopic appendectomy in 52% patients; male (54.5%); median age of 83 yrs in elderly group and 29.5 yrs in non-elderly group; ASA I/II (91%)	Elderly: ≥75 years Non-elderly: <75 years	2,060 (elderly: 65; non-elderly: 1,995)	*Complicated appendicitis (perforation or gangrene/necrotic)*: RR 4.86 (95% CI: 3.91, 6.04) *Postoperative intra-abdominal abscess*: RR 2.63 (95% CI: 0.83, 8.33) *Overall post-operative complication:* RR 5.72 (95% CI: 4.23, 7.73) *Post-operative wound infection*: RR 5.90 (95% CI: 3.14, 11.08) *Use of laparoscopic appendectomy*: RR 0.88 (95% CI: 0.68, 1.16) *Conversion rate* (from laparoscopy to open surgery): RR 5.63 (95% CI: 3.11, 10.18) *Mortality (30-day):* RR 40.92 (95% CI: 9.35, 179.14)
Pokharel et al. (5)	Retrospective review	Nepal	Patients undergoing open appendectomy (94.5%) due to acute appendicitis; male (40% in elderly and 33.3% in non-elderly group)	Elderly: ≥60 years Non-elderly: <60 years	200 (elderly: 50; non-elderly: 150)	*Postoperative intra-abdominal abscess*: RR 1.50 (95% CI: 0.14, 16.2) *Overall post-operative complication:* RR 2.00 (95% CI: 1.21, 3.31) *Post-operative wound infection*: RR 2.73 (95% CI: 1.23, 6.04) *Use of laparoscopic appendectomy*: RR 2.50 (95% CI: 0.80, 7.84) *Mortality (30-day):* RR 14.8 (95% CI: 0.72, 303.3) *Length of hospital stay (mean, SD; in days):* 5.3 (2.6) vs. 2.2 (1.7)
Zbierska et al. (23)	Retrospective review	Poland	Patients undergoing appendectomy due to acute appendicitis; majority undergoing laparoscopic appendectomy (53.2%); female (56%); mean age of 71.6 yrs in elderly group and 32.4 yrs in non-elderly group	Elderly: ≥65 years Non-elderly: <65 years	274 (elderly: 23; non-elderly: 251)	*Complicated appendicitis (perforation or gangrene/necrotic)*: RR 1.86 (95% CI: 1.28, 2.70) Overall post-operative complication*: RR 1.62 (95% CI: 0.62, 4.22) *Use of laparoscopic appendectomy*: RR 0.44 (95% CI: 0.20, 0.96) *Conversion rate* (from laparoscopy to open surgery): RR 0.32 (95% CI: 0.02, 5.14) *Length of hospital stay (mean, SD; in days):* 6.08 (5.04) vs. 4.69 (3.4) *Operative time (mean, SD; in minutes):* 78.3 (41.9) vs. 79 (27.5) No deaths in either of the two groups *included hematoma of wound, wound dehiscence, partial/total bowel obstruction, exanthema, intraabdominal abscess
Guller et al. (24)	Retrospective analysis of NIS data (NIS is publicly available database in USA)	USA	Patients undergoing laparoscopic appendectomy due to acute appendicitis; mean age of 72.5 yrs in elderly group and 29.8 yrs in non-elderly group; female (52.9% in elderly and 48.5% in non-elderly group)	Elderly: ≥65 years Non-elderly: <65 years	32,406 (elderly: 1,475; non-elderly: 30,931)	*Complicated appendicitis (perforation or gangrene/necrotic)*: RR 2.05 (95% CI: 1.92, 2.18) *Overall post-operative complication:* RR 2.78 (95% CI: 2.50, 3.09) *Length of hospital stay (mean, SD; in days):* 4.16 (0.18) vs. 2.37 (0.03) *Post-operative wound infection/complication*: RR 2.25 (95% CI: 1.18, 4.32)
Guller et al. (24)	Retrospective analysis of NIS data (NIS is publicly available database in USA)	USA	Patients undergoing open appendectomy due to acute appendicitis; mean age of 73.9 yrs in elderly group and 27.8 yrs in non-elderly group; female (48.7% in elderly and 38.6% in non-elderly group)	Elderly: ≥65 years Non-elderly: <65 years	112,884 (elderly: 8,001; non-elderly: 104,883)	*Complicated appendicitis (perforation or gangrene/necrotic)*: RR 1.97 (95% CI: 1.93, 2.01) *Overall post-operative complication:* RR 3.03 (95% CI: 2.92, 3.15) *Length of hospital stay (mean, SD; in days):* 7.13 (0.09) vs. 3.42 (0.03) *Post-operative wound infection/complication*: RR 5.33 (95% CI: 4.34, 6.53) *Mortality*: RR 34.0 (95% CI: 26.0, 44.4)
Ghnnam et al. (25)	Retrospective analysis of medical records	Egypt	Patients undergoing open appendectomy (?) due to acute appendicitis; mean age of 74.9 yrs in elderly group and 23.2 yrs in non-elderly group; female (52.2% in elderly and 27.5% in non-elderly group)	Elderly: ≥60 years Non-elderly: <60 years	63 (elderly: 23; non-elderly: 40)	*Length of hospital stay (mean, SD; in days):* 7.2 (4.6) vs. 2.2 (0.46) *Operative time (mean, SD; in minutes):* 86 (26) vs. 56 (18) *Post-operative wound infection/complication*: RR 5.22 (95% CI: 1.15, 23.75) *Complicated appendicitis (perforation or gangrene/necrotic)*: RR 3.47 (95% CI: 1.77, 6.84) *Overall post-operative complication:* RR 6.95 (95% CI: 1.61, 30.0)
Sammut et al. (26)	Retrospective review	Malta	Patients undergoing appendectomy due to acute appendicitis; majority with open appendectomy (54%); female (72.7% in elderly and 48.6% in non-elderly group)	Elderly: >75 years Non-elderly: ≤75 years	173 (elderly: 33; non-elderly: 140)	*Length of hospital stay (mean, SD; in days):* 15.81 (11.2) vs. 5.98 (11.1) *Overall post-operative complication:* RR 1.59 (95% CI: 1.08, 2.34) *Mortality*: RR 4.24 (95% CI: 0.27, 66.1) *Use of laparoscopic appendectomy*: RR 1.25 (95% CI: 0.87, 1.80)

There was heterogeneity with regard to the cut-offs used by the included studies to define “elderly.” A total of 7 studies used the cut-off of ≥ 65 years to label subjects as elderly, whereas 4 studies used the cut-off of ≥ 60 years. Two studies used ≥ 75 years as cut-off, and one study each used the cut-off of ≥ 68 and ≥ 70 years, respectively (Table 1). In 7 studies, laparoscopic appendectomy was done, whereas, in 3 studies, open appendectomy was done. In the remaining 6 studies, the procedure used was mixed with some receiving open appendectomy and some laparoscopic appendectomy. The results of the quality evaluation of the included studies are provided in Supplementary Tables 2, 3. The included studies were of modest to good quality.

**Figure 2:**
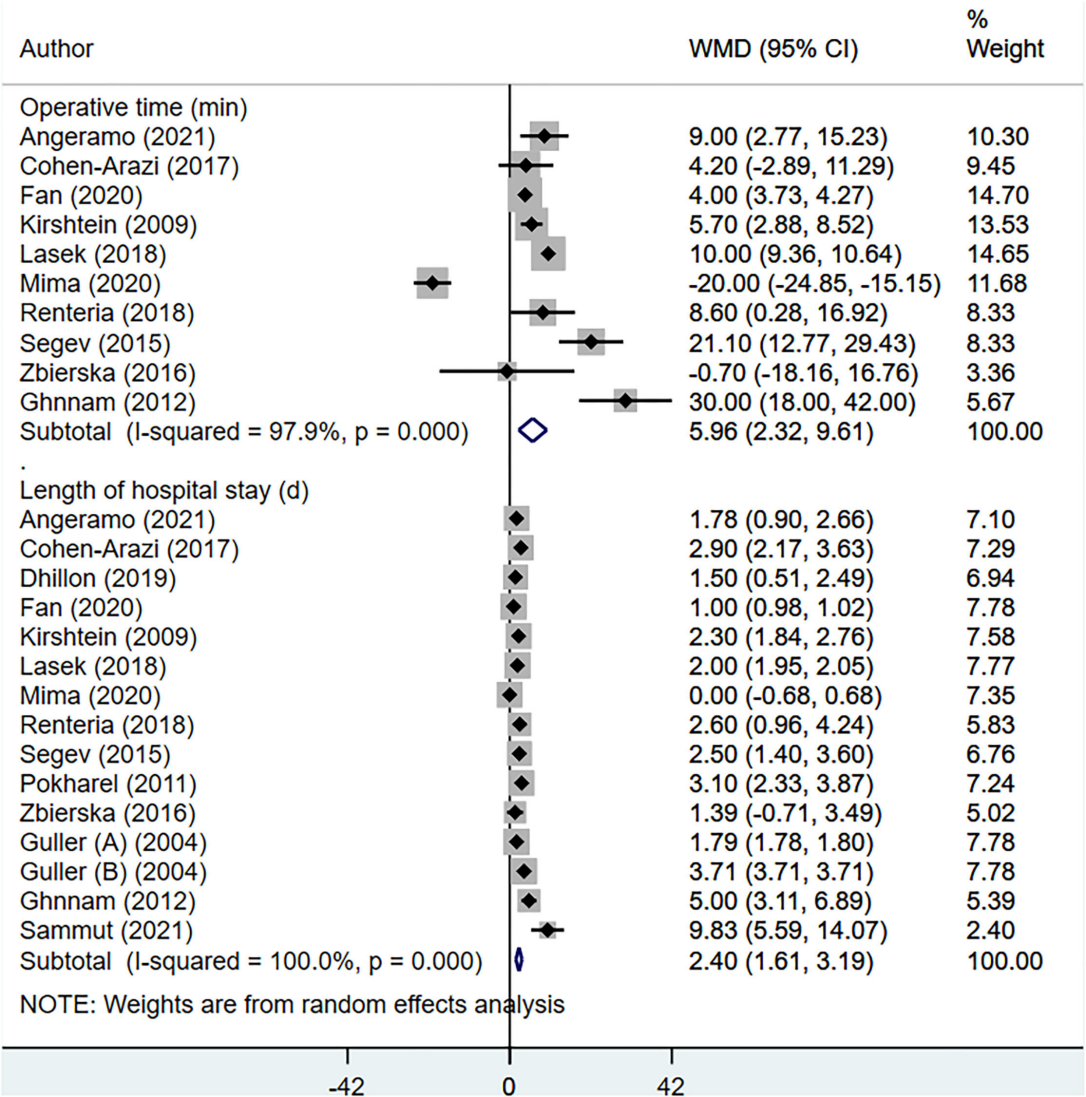
Operative time and length of hospital stay among elderly subjects, compared to non-elderly subjects.

**Figure 3:**
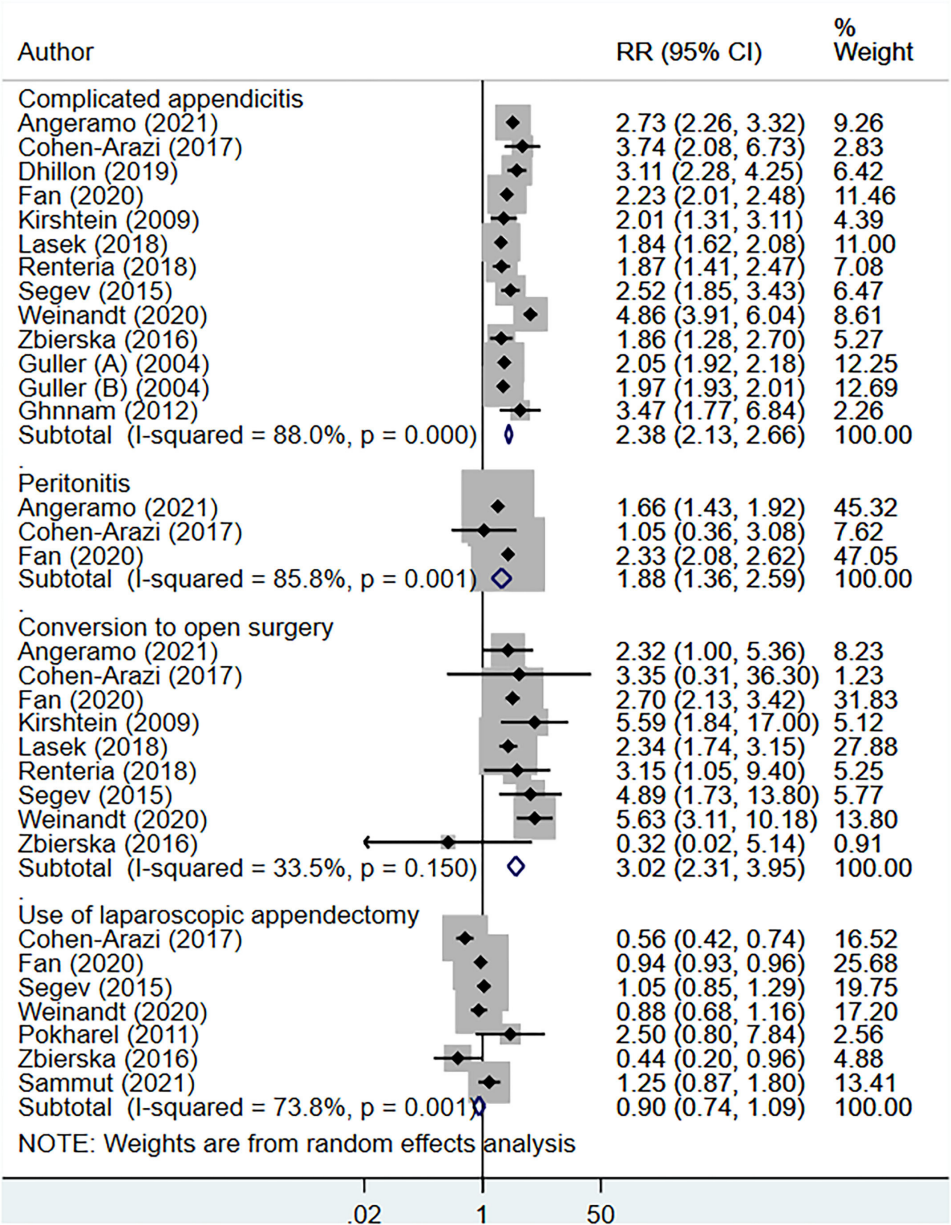
Clinical outcomes of appendectomy among elderly subjects, compared to non-elderly subjects.

**Figure 4:**
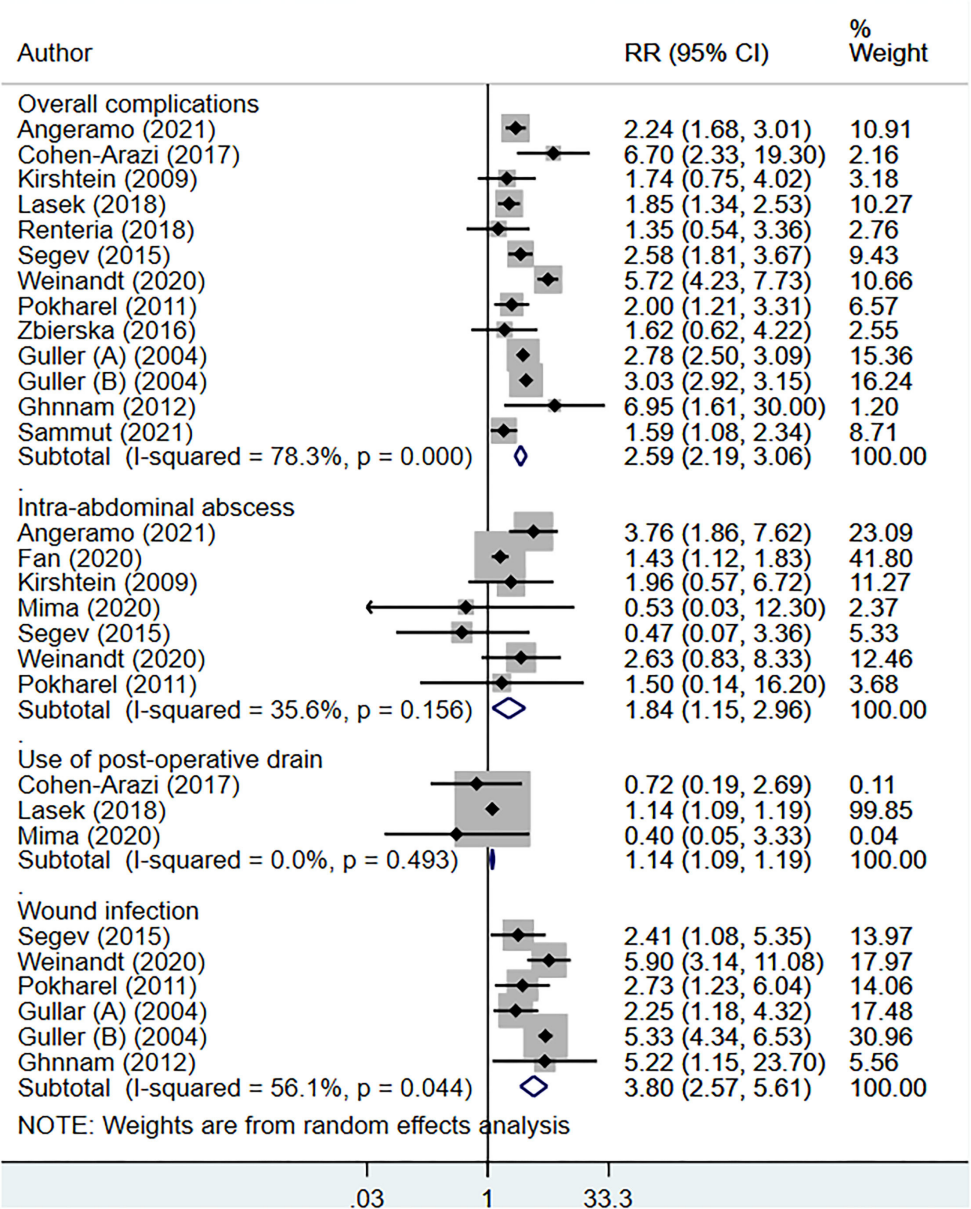
Postoperative complications of appendectomy among elderly subjects, compared to non-elderly subjects.

**Figure 5:**
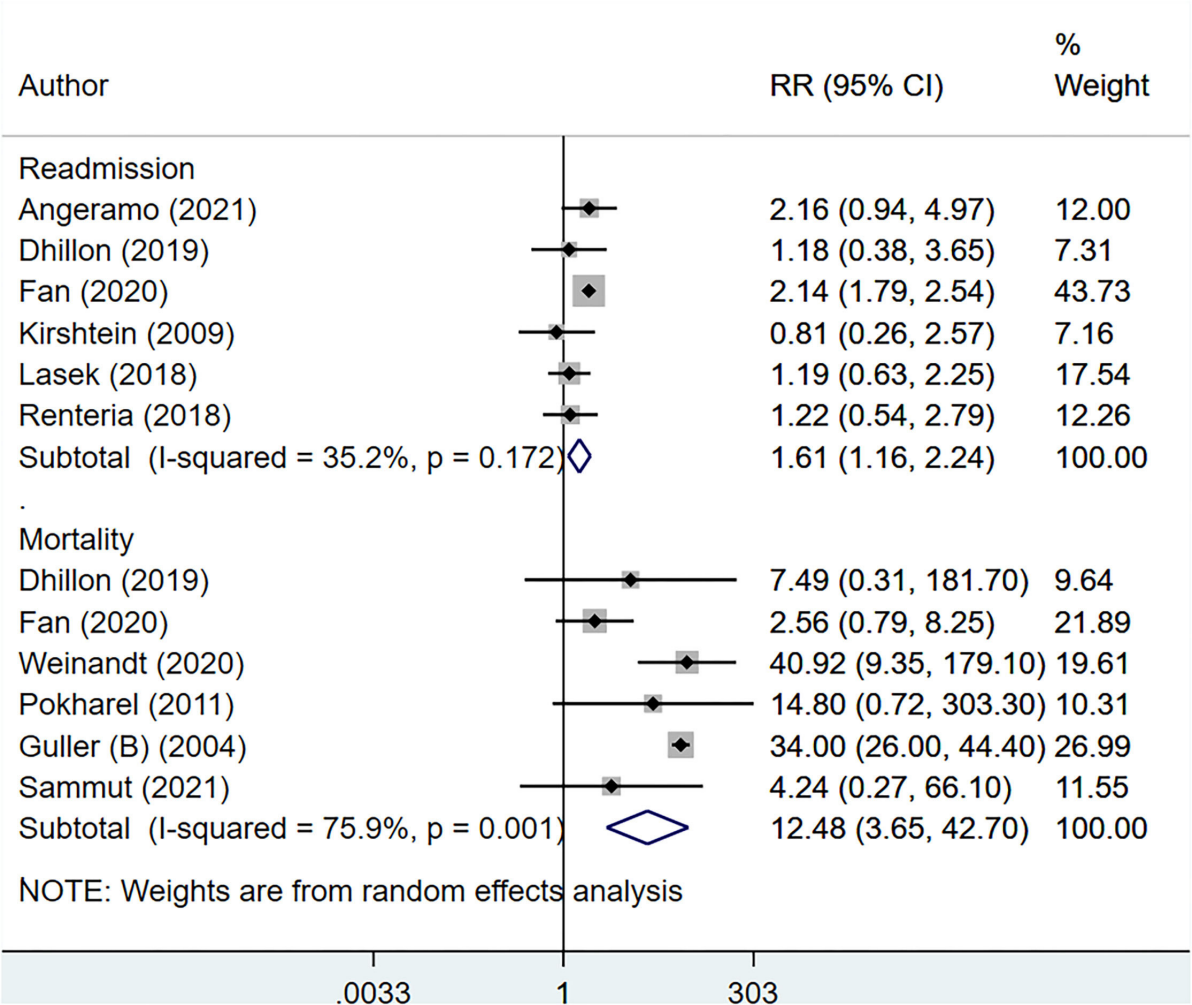
Risk of readmission and mortality among elderly subjects, compared to non-elderly subjects.

The total operative time (in minutes) [WMD, 5.96; 95% CI: 2.32, 9.61, *I*^2^ = 97.9%, *N* = 10], and the length of hospital stay (in days) [WMD, 2.40; 95% CI: 1.61, 3.19, *I*^2^ = 100.%, *N* = 15] was higher for elderly, compared to younger patients (Figure 2). Compared to non-elderly patients, those that were elderly had increased risk of complicated appendicitis (perforation, gangrene or necrosis) [RR, 2.38; 95% CI: 2.13, 2.66, *I*^2^ = 88.%, *N* = 13], peritonitis [RR, 1.88, 95% CI: 1.36, 2.59, *I*^2^ = 85.8%, *N* = 3] and conversion from laparoscopic to open appendectomy [RR, 3.02, 95% CI: 2.31, 3.95, *I*^2^ = 33.5%, *N* = 9] (Figure 3). No significant difference in the overall use of laparoscopic appendectomy [RR, 0.90; 95% CI: 0.74, 1.09, *I*^2^ = 73.8%, *N* = 7] among elderly and non-elderly subjects was noted (Figure 3). The risk of overall postoperative complications [RR, 2.59; 95% CI: 2.19, 3.06, *I*^2^ = 78.3%, *N* = 13], intra-abdominal abscess [RR, 1.84; 95% CI: 1.15, 2.96, *I*^2^ = 35.6%, *N* = 7], wound infection [RR, 3.80, 95% CI: 2.57, 5.61, I^2^ = 56.1%, *N* = 6], and use of postoperative drainage [RR, 1.14; 95% CI: 1.09, 1.19, *I*^2^ = 0.0%, *N* = 3] was higher among the elderly (Figure 4). Similarly, the risk of readmission (30 days) [RR, 1.61; 95% CI: 1.16, 2.24, *I*^2^ = 35.2%, *N* = 6] and mortality (30 days) [RR, 12.48; 95% CI: 3.65, 42.7, *I*^2^ = 75.9%, *N* = 6] were higher among elderly, compared to younger subjects (Figure 5). For all the outcomes analyzed, there was no evidence of publication bias (*p* > 0.05).

Subgroup analysis base showed that, irrespective of the type of appendectomy (i.e., laparoscopic, open, and mixed), elderly subjects, compared to younger subjects, had an increased risk of complicated appendicitis, overall postoperative complication, wound infection, and length of hospital stay (in days) (Table 2). The risk of conversion to open appendectomy [RR, 2.61; 95% CI: 2.19, 3.11, *I*^2^ = 0%, *N* = 5], peritonitis [RR, 1.97; 95% CI: 1.42, 2.75, *I*^2^ = 92.1%, *N* = 2], intra-abdominal abscess [RR, 1.96; 95% CI: 1.03, 3.74, *I*^2^ = 57.5%, *N* = 4], use of postoperative drainage [RR, 1.14; 95% CI: 1.09, 1.19, *I*^2^ = 0%, *N* = 2] and readmission [RR, 1.63; 95% CI: 1.13, 2.34, *I*^2^ = 42.3%, *N* = 5] was increased in elderly subjects undergoing laparoscopic appendectomy, compared to non-elderly subjects with laparoscopic appendectomy. The risk of mortality was increased among elderly subjects in open appendectomy [RR, 33.8; 95% CI: 25.9, 44.1, *I*^2^ = 0.0%, *N* = 2] but not in laparoscopic appendectomy. Similarly, the operative time (in minutes) was increased in elderly subjects with open appendectomy [WMD, 3.; 95% CI: 18, 24, *N* = 1] but not in laparoscopic appendectomy (Table 2).

**Table 2:** Outcomes in elderly, compared to non-elderly, based on the type of appendectomy.

**Outcomes**	**Laparoscopic**	**Open**	**Mixed^†^**
	**Pooled effect size (95% CI); (*****N*** **= total number of studies; *I*^2^)**		
Complicated appendicitis	RR 2.11 (1.91, 2.33); (*N =* 6; *I*^2^ = 63.9 %) ^*^	RR 2.35 (1.41, 3.94); (*N =* 2; *I*^2^ = 62.9 %)^*^	RR 3.06 (2.12, 4.41); (*N =* 5; *I*^2^ = 83.6 %)^*^
Peritonitis	RR 1.97 (1.42, 2.75); (*N =* 2; *I*^2^ = 92.1%)^*^	----	RR 1.05 (0.36, 3.07); (*N =* 1)
Conversion to open surgery	RR 2.61 (2.19, 3.11); (*N =* 5; *I*^2^ = 0.0%)^*^	----	RR 4.35 (2.15, 8.81); (*N =* 4; *I*^2^ = 25.4 %)^*^
Overall complication	RR 2.22 (1.74, 2.82); (*N =* 5; *I*^2^ = 59.1%)^*^	RR 2.81 (1.94, 4.07); (*N =* 3; *I*^2^ = 48.0%)^*^	RR 2.98 (1.62, 5.46); (*N =* 5; *I*^2^ = 87.5%)^*^
Post-operative intra-abdominal abscess	RR 1.96 (1.03, 3.74); (*N =* 4; *I*^2^ = 57.5%)^*^	RR 1.50 (0.14, 16.2); (*N =* 1)	RR 1.33 (0.26, 6.94); (*N =* 1)
Post-operative wound infection	RR 2.25 (1.18, 4.31); (*N =* 1)^*^	RR 4.71 (3.23, 6.87); (*N =* 3; *I*^2^ = 21.5%)^*^	RR 3.91 (1.63, 9.37); (*N =* 2; *I*^2^ = 66.3%)^*^
Use of post-operative drain	RR 1.14 (1.09, 1.19); (*N =* 2; *I*^2^ = 0.0%) ^*^	----	RR 0.72 (0.19, 2.71); (*N =* 1)
Readmission	RR 1.63 (1.13, 2.34); (*N =* 5; *I*^2^ = 42.3%) ^*^	----	RR 1.18 (0.38, 3.66); (*N =* 1)
Mortality	RR 2.56 (0.79, 8.27); (*N =* 1)	RR 33.8 (25.9, 44.1); (*N =* 2; I^2^= 0.0%) ^*^	RR 17.7 (4.2, 75.3); (*N =* 3; I^2^= 19.6%) ^*^
Operative time (min)	WMD 2.92 (−1.24, 7.09); (*N =* 6; *I*^2^ = 98.7%)	WMD 30.0 (18.0, 24.0); (*N =* 1) ^*^	WMD 9.21 (−4.15, 22.6); (*N =* 3; *I*^2^ = 81.5%)
Length of hospital stay (days)	WMD 1.57 (1.13, 2.02); (*N =* 7; *I*^2^ = 99.9%)^*^	WMD 3.64 (3.05, 4.22); (*N =* 3; *I*^2^ = 52.5%)^*^	WMD 2.66 (1.42, 3.90); (*N =* 5; *I*^2^ = 77.3%)^*^

*RR, relative risk; WMD, weighted mean difference;*

* *statistically significant at p < 0.05;*

† *indicates that the patient population was heterogenous with respect to type of appendectomy, i.e., some receiving open appendectomy and others receiving laparoscopic appendectomy*.

## Discussion

The current meta-analysis was conducted to compare the outcomes of appendectomy among elderly and non-elderly subjects. Additionally, it also aimed to understand if the outcomes differ by the type of appendectomy; i.e., laparoscopic or open. The study found an increased risk of adverse clinical outcomes among elderly subjects, and this was largely irrespective of the type of appendectomy. The total operative time and the length of hospital stay were higher for the elderly. Compared to non-elderly patients, those that were elderly had an increased risk of complicated appendicitis, peritonitis, postoperative complications, intra-abdominal abscess, wound infection, and conversion from laparoscopic to open appendectomy. The risk of readmission and mortality within 30 days of operation was also higher in elderly subjects.

A recent systematic review and meta-analysis by Wang et al. documented that, among the elderly subjects, postoperative mortality was lower following laparoscopic appendectomy (8). Also, postoperative complications and wound infections were reduced following the laparoscopic procedures. The intra-abdominal abscess was similar between both open and laparoscopic appendectomy. Based on their findings, the authors suggested laparoscopic appendectomy to be safe and feasible for the elderly (8). While this may be true, our review presents a slightly different perspective on it. Our findings suggest that, while in the elderly, laparoscopic appendectomy may be better than open appendectomy, yet, the outcomes when compared to non-elderly subjects are sub-optimal. Therefore, irrespective of the mode of surgical management, the treating surgical team should be more careful when dealing with elderly subjects. The World Society of Emergency Surgery (WSES), Jerusalem also recommends that laparoscopic appendectomy is a safe and effective method to treat AA in the elderly and the obese (27).

In our review, we noted an increased risk of complicated appendicitis such as perforation or gangrene among elderly subjects. This is possibly attributed to the delayed presentation (28). One commonly reported reason for such delay is the lack of a classical triad of pain (lower quadrant), fever, and leucocytosis (29, 30). Studies have shown that the classical trial is present only in less than a fourth of the elderly patients (29, 30). Previous studies have reported a delay of at least 48 h in around a third of the elderly patients (31, 32). To overcome this issue, clinicians have now started to develop appendicitis risk prediction models in adults (33, 34). The Appendicitis Inflammatory Response (AIR) score and the Adult Appendicitis Score (AAS) score are judged to be the best performing clinical prediction scores and are noted to have substantial discriminating power in adults with suspected acute appendicitis (27). Another proposed reason for the increased risk of perforation and necrosis is the decrease in the lymphoid tissue as well as blood supply in the elderly (35). Elderly patients with complicated appendicitis are less likely to respond to conservative management. The prime reason is that elderly subjects have comparatively less physiological reserve than younger subjects (36). Also, as they are frailer, the window period to intervene is less. There is evidence to show that surgical management is superior to conservative management for patients presenting with complicated appendicitis (37). The review found an increased hospital stay among the elderly. This could be due to the increased risk of postoperative complications, which necessitate a long admission. It could also be due to the increased risk of conversion from laparoscopic appendectomy to open appendectomy. The increased risk of readmission observed in the meta-analysis could be due to associated comorbidities in the elderly as well as associated increased length of hospital stay. A study by Moghadamyeghaneh et al. found that the risk of unplanned readmissions in the elderly was higher in those that had higher hospital stays (38). The likely reason for the same was the higher rates of comorbidities and increased postoperative complications noted in the elderly.

The meta-analysis documented an increased risk of peri- and postoperative adverse outcomes in elderly subjects, compared to non-elderly subjects. Due to this increased risk, non-surgical/conservative management may be more advisable for elderly subjects but considering that the risk of life-threatening complications, such as complicated appendicitis (perforation, gangrene, or necrosis) and generalized peritonitis, is more common in the elderly, surgical approach is still the advisable management strategy. The results of this meta-analysis can impact the clinical practice and underscore the need for an enhanced vigilance and awareness of the surgeon regarding the non-specific presentation of acute appendicitis in the elderly. This might lead to an early diagnosis and management in this high-risk group. Furthermore, this could alleviate the risk of peri-operative complications and possibly reduce the need for conversion from laparoscopic to open surgery. The findings also support the need for better peri- and postoperative care for elderly subjects undergoing appendectomy and emphasize that the surgeon should be careful while treating the elderly, irrespective of the mode of appendectomy, i.e., open or laparoscopic.

There could be differences in the outcomes across the included studies based on the services available at the treating hospital, the skills of the surgeon, and the quality of post-operative care provided. This could be one of the factors leading to a moderate degree of heterogeneity noted for some of the outcomes. Furthermore, the included studies did not mention clearly whether the estimates presented in their study were accounted or adjusted for the baseline differences in the elderly and non-elderly subjects. This is important to consider as, in some of the included studies, there were baseline differences, particularly concerning the presence of comorbidities, and these could affect the outcome of the surgery. All the included studies were observational, and, of them, mostly used retrospectively collected data. The possibility that important confounders are not adjusted in the analysis cannot be ruled out. The included studies were from a diverse geography and possibly involved surgical teams with varied skills, techniques, and experience. These also may have contributed to the heterogeneity.

## Data Availability Statement

Publicly available datasets were analyzed in this study. This data can be found at: PubMed, Scopus and Google academic databases from inception until October 2021 for relevant publications.

## Author Contributions

JY conceived and designed the study. JY, WH, LY, and QC were involved in literature search and data collection. QC, WH, and LY analyzed the data. JY and XL wrote the paper. XL reviewed and edited the manuscript. All authors read and approved the final manuscript.

## Conflict of Interest

The authors declare that the research was conducted in the absence of any commercial or financial relationships that could be construed as a potential conflict of interest.

## Publisher's Note

All claims expressed in this article are solely those of the authors and do not necessarily represent those of their affiliated organizations, or those of the publisher, the editors and the reviewers. Any product that may be evaluated in this article, or claim that may be made by its manufacturer, is not guaranteed or endorsed by the publisher.
